# Fatty Acid Elongase 7 (ELOVL7) Plays a Role in the Synthesis of Long-Chain Unsaturated Fatty Acids in Goat Mammary Epithelial Cells

**DOI:** 10.3390/ani9060389

**Published:** 2019-06-25

**Authors:** Hengbo Shi, Li Wang, Jun Luo, Jianxin Liu, Juan. J. Loor, Hongyun Liu

**Affiliations:** 1 Institute of Dairy Science, College of Animal Sciences, Zhejiang University, Hangzhou 310058, China; shihengbo@zstu.edu.cn (H.S.); lylian@zju.edu.cn (L.W.); liujx@zju.edu.cn (J.L.); 2 Zhejiang Provincial Key Laboratory of Silkworm Bioreactor and Biomedicine, College of Life Sciences and Medicine, Zhejiang Sci-Tech University, Hangzhou 310018, China; 3 College of Animal Science and Technology, Northwest A&F University, Yangling 712100, China; luojun@nwsuaf.edu.cn; 4 Mammalian NutriPhysioGenomics, Department of Animal Sciences and Division of Nutritional Sciences, University of Illinois, Urbana, IL 61801, USA

**Keywords:** fatty acid elongation, substrate, unsaturated fatty acid, signaling molecule

## Abstract

**Simple Summary:**

Very-long-chain elongases are required for the synthesis of essential fatty acids in non-ruminants. Whether the fatty acid elongase 7 (ELOVL7) plays a role in ruminants is unclear. We demonstrated, in goat mammary epithelial cells, that ELOVL7 activation resulted in greater concentrations of vaccenic (C18:1n7) and linoleic (C18:2) acid, and lower concentrations of palmitoleic (C16:1n7) and oleic (C18:1n9) acid. Knockdown of *ELOVL7* increased the concentration of C18:1n9. The data support a novel role of ELOVL7 in altering long-chain unsaturated fatty acids in goat mammary epithelial cells.

**Abstract:**

In humans, fatty acid elongase 7 (ELOVL7) plays a role in synthesis of long-chain saturated fatty acids. Whether ELOVL7 protein plays a role in ruminants is unclear. The transcript abundance of ELOVL7 in goat mammary tissue was assessed at three stages of lactation. Results showed that *ELOVL7* had the highest expression in the dry period compared with peak and late lactation period. Results revealed that *ELOVL7* overexpression was correlated with lower expression in diacylglycerol O-acyltransferase 2 (*DGAT2*) and stearoyl-CoA desaturase 1 (*SCD1*), and had no significant effect on triacylglycerol concentration. Overexpression of *ELOVL7* significantly decreased the concentration of palmitoleic (C16:1n7) and oleic (C18:1n9) acid, and increased the concentration of vaccenic (C18:1n7) and linoleic (C18:2) acid. Overexpression of *ELOVL7* significantly upregulated the elongation index of C16:1 in goat epithelial mammary cells (GMEC), but had a minor effect on that of palmitate (C16:0). Knockdown of *ELOVL7* decreased mRNA expression of fatty acid binding protein 3 (*FABP3*) and fatty acid desaturase 2 (*FADS2*) and had a minor effect on triacylglycerol concentration; however, it increased concentration of C18:1n9 in GMEC. The elongation indices of C16:0 and C16:1 did not differ due to knockdown of *ELOVL7.* The minor change for the C16:0 and stearate (C18:0) was observed after activation of ELOVL7, suggesting the two fatty acids are not the preferential substrates of ELOVL7 in cultured GMEC. However, changes in C18:1n9 and C18:2 after overexpression or knockdown of *ELOVL7* indicated a biological functional role of ELOVL7. Collectively, our data highlighted a role of ELOVL7 in long-chain unsaturated fatty acid elongation in goat mammary epithelial cells.

## 1. Introduction

Milk fat is an important energy source in terms of dairy production. In ruminants, about half of the milk fats are synthesized de novo [1]. The de novo synthesis of fatty acids (FAs) involves many enzymes containing acetyl-CoA carboxylase (ACC) and fatty acid synthase (FASN). The remaining FAs come from diet and adipose tissue mobilization [1]. FA elongases and desaturases cooperate to synthesize monounsaturated or polyunsaturated fatty acids [2,3]. It is well investigated that the FA desaturase enzymes (e.g., stearoyl-CoA desaturase 1 (SCD1)) catalyze the conversion of saturated FAs (SFAs) to monounsaturated FAs (MUFAs) or polyunsaturated FAs (PUFAs) in ruminants [3,4,5]. Early in vitro studies showed that the FA elongase enzyme regulated the chain length and the degree of unsaturation of FAs [6]. Recent data in the ruminant mammary cells underscored the crucial role of some FA elongases in extending long-chain fatty acids (LCFAs) [7,8]. 

The FA elongase, also named elongation of very-long-chain fatty acid-like fatty acid elongase (ELOVL), catalyzes the rate-limiting step of the LCFA elongation cycle. The ELOVL family were identified in mammalian tissue (from ELOVL1 to ELOVL7). They display tissue-specific expression and exhibit characteristic substrate specificities in vitro [9]. An in vitro study in human embryonic kidney (HEK) 293T cells showed that ELOVL1 and 4 was the most potent elongase for C22:0- and C24:0-CoAs, and are thus linked to C24 sphingolipid synthesis [9]. ELOVL2 and 5 acted specifically toward polyunsaturated acyl-CoAs. The activity of ELOVL6 was extremely high toward palmitoyl-CoA (C16:0). Both ELOVL3 and 7 exhibited activities toward C16–C22 acyl-CoAs [6]. Previous data showed that ELOVL5, 6, and 7 were expressed in goat lactating mammary gland tissue [10]. FA profile analysis in goat epithelial mammary cells (GMEC) confirmed that ELOVL6 plays a role in the elongation of long-chain saturated FA (SFA)(C16:0 to stearate (C18:0) [8]) and ELOVL5 plays a role in the elongation of palmitoleic (C16:1n7) and oleic (C18:1n9) acid [7]. However, compared with ELOVL5 and 6, the precise role of ELOVL7 have not been determined in mammary gland tissue. 

ELOVL7 was reportedly involved in prostate cancer growth via controlling the synthesis of LCFA [9]. Knockdown of *ELOVL7* in prostate cancer cell lines resulted in reduced levels of saturates FA [11]. Biochemical characterization analysis revealed that ELOVL7 exhibited high activity toward acyl-CoAs with 18-carbon chain length, and plays a role in the elongation of SFAs with up to 24 carbons [9,12]. As well as other lipogenic enzymes, ELOVL7 activity was regulated through sterol regulatory element-binding transcription factor 1 (SREBP1) and mammalian target of rapamycin (mTOR) pathways [11,13]. Recent data showed that *ELOVL7* is expressed in goat [10] and cow [14] mammary tissue. The evidence in bovine mammary cells that the α-linolenic acid (18:3n-3) enhanced *ELOVL7* promoter activity and resulted in the accumulation of cellular fat [14], suggests a role of ELOVL7 in FA metabolism. Collectively, these findings emphasize the important role of ELOVL7 controlling the FA synthesis.

The recent demonstration that *ELOVL7* is expressed throughout mammary epithelial cell differentiation [15] is suggestive of a role of ELOVL7 in fatty acid elongation in the mammary gland tissue. However, whether ELOVL7 is essential for the synthesis and alteration of LCFA composition in ruminant mammary epithelial cells (MECs) remains to be determined. Here, it is hypothesized that ELOVL7 has a role in the process of LCFA synthesis in GMEC. To assess the role of ELOVL7, both adenoviral-mediated overexpression and RNA interference were performed in GMEC cultures. Our results illustrated that alteration of ELOVL7 changed the lipogenic gene expression and FA profile in GMEC and highlighted an important role for ELOVL7 in LCFA elongation. 

## 2. Materials and Methods 

### 2.1. Transcript Abundance of ELOVL7 in Goat Mammary Gland Tissue

Dairy goat (Xinong Saanen breed) mammary gland tissue at peak lactation (100 days postpartum), late-lactation (cessation of milking, 310 days postpartum), and the non-lactating period were collected, and the transcriptome were sequenced (each period collected samples from three goats) [10]. All animal collection and utility protocols were approved by the Animal Care and Use Committee of the Northwest A&F University. The transcriptome dataset from goat mammary tissue was deposited at National Center for Biotechnology Information (NCBI) (BioProject ID: PRJNA243005). The expression of *ELOVL7* was measured by reads per kilobase of exon model per million mapped reads (RPKM).

### 2.2. Adenovirus Generation 

The whole process for generation and proliferation of recombinant adenovirus expressing *ELOVL7* (Ad-ELOVL7) was carried out as previously described [16]. 

### 2.3. Cell Culture

GMEC were isolated from peak lactation Xinong Saanen goats as previously described [17,18]. Details of cell culture were described recently [19,20]. The 293A cells for adenovirus generation were cultured in basal DMEM medium (Gibco Invitrogen, Carlsbad, CA, USA) containing 10% fetal bovine serum and penicillin/streptomycin (10K unit/L, Harbin Pharmaceutical Group, China). 

The recombinant adenovirus expressing green fluorescent protein (Ad-GFP) was used as a control. The GMEC were transfected with adenovirus supernatant (Ad-ELOVL7 or Ad-GFP) at about 80% confluence. The transfected GMEC were collected after 48 h of culture for lipid extraction, total RNA extraction, and TAG assay. 

### 2.4. RNA Interference

For *ELOVL7* mRNA interference, cells cultured in six-well plates were incubated with 50 nM siRNA using transfection reagent (Lipofectamine® RNAiMAX, Thermo Fisher Scientific Inc., Waltham, MA, USA) in medium without antibiotic. The transfection was performed according to manufacturer’s instructions. Transfected GMEC were harvested at 48 h for RNA extraction, TAG assays, and FA extraction. The siRNA for *ELOVL7* (Accession: XM_005694673.2) (siELOVL7) were designed and synthesized by Jima Biotechnology Co., Ltd. (Shanghai, China). The sequences for the siELOVL7 are described in Table S1. A functional non-targeting siRNA was used as a control (siNC). 

### 2.5. Total RNA Extraction and Quantitative Real-Time PCR

RNA Prep Pure Cell Kit was used to extract the total RNA from GMEC (Tiangen Biotech Co. Ltd., Beijing, China). Genomic DNA contamination was removed using DNase provided with the kit. Synthesis of cDNA was used the PrimeScript^TM^ RT Reagent Kit with (Takara Bio Inc., Otsu, Japan). The quantitative real-time PCR (qPCR) was performed using SYBR Green (SYBR^®^ Premix Ex Taq™ II, Perfect Real Time, Takara Bio Inc.). 

Several genes related to TAG synthesis (diacylglycerol O-acyltransferase 1 (*DGAT1*), *DGAT2*, perilipin2 (*PLIN2*)), or *SCD1*, fatty acid desaturase 1 (*FADS1*) and *FADS2* de novo synthesis (*ACC* and *FASN*), and FA transport (fatty acid binding protein 3 (*FABP3*)) were selected to evaluate functional outcomes after alteration of *ELOVL7* expression. All the qPCR reactions were performed in a Bio-Rad CFX96 (Bio-Rad Laboratories Inc., Hercules, CA) sequence detector, and data were normalized using the geometric mean of the 3 reference genes, ubiquitously expressed transcript (*UXT*), mitochondrial ribosomal protein L39 (*MRPL39*), and ribosomal protein S9 (*RPS9*) [21]. The primer sequences of the genes are described in Table S2.

### 2.6. FA Analysis

Total lipid extraction and methylation were performed according to Shi et al. [22]. The FA profile was analyzed using a gas chromatography–mass spectrophotometer 7890A (Agilent Technologies, Santa Clara, CA, USA) installed with a DB-23 column following a published procedure [23]. 

### 2.7. Statistical Analysis

Treatments were replicated at least 3 times in culture wells, and the qPCR was performed in triplicate. Data of qPCR were analyzed using the 2^−ΔΔCt^ method. The elongation index was calculated as the ratio between the elongation products and the substrates. For example, an elongation index of C16:0 = (C18:0 + C18:1n-9)/C16:0, an elongation index of C16:1 = C18:1n-7/C16:1. Significance of RNA expression of *ELOVL7* in mammary gland tissue across different stages of lactation was determined by one-way ANOVA. All of the data are expressed as mean ± standard error of the mean. The data for qPCR, triacylglycerol (TAG) content, elongation index, and FA ratio after altering *ELOVL7* expression, were determined via Student’s t-test. Significance was declared at *p* < 0.05.

## 3. Results

### 3.1. ELOVL7 is Expressed in Dairy Goat Mammary Gland Tissue

**Figure 1:**
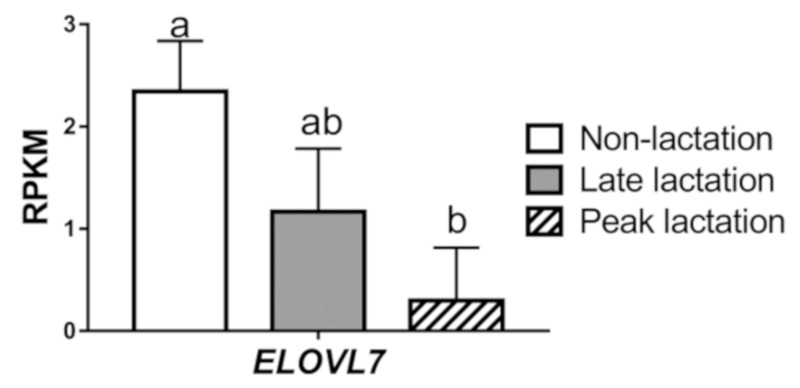
The mRNA abundance of elongation of very-long-chain fatty acid-like fatty acid elongase 7 (*ELOVL7*) in goat mammary gland tissues. Values are means ± SEM for 3 individual goats. Significance of RNA expression of *ELOVL7* in mammary gland tissue across different stages of lactation was determined by one-way ANOVA. The different letters denote significant (*p* < 0.05) differences.

As shown in Figure 1, the non-lactating mammary gland had the highest RPKM value for *ELOVL7*, followed by the late-lactation samples (*p* > 0.05). The transcript of *ELOVL7* was lowest at peak lactation among the three stages (*p* < 0.01, non-lactation vs. peak lactation). 

### 3.2. Overexpression of ELOVL7 Downregulated Genes Related to TAG Synthesis and FA Desaturation

**Figure 2:**
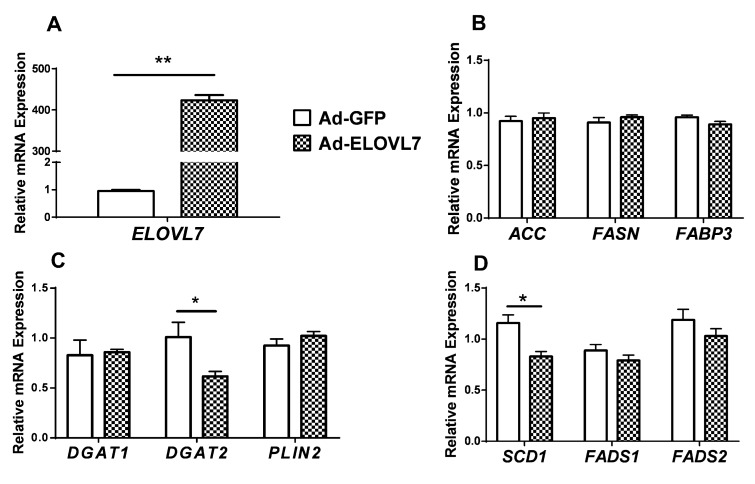
Overexpression of elongation of very-long-chain fatty acid-like fatty acid elongase 7 (*ELOVL7*) altered gene expression. Goat mammary epithelial cells (GMEC) were transfected with Ad-ELOVL7 or Ad-GFP for 48 h and then collected for mRNA extraction. Panel A: mRNA expression of *ELOVL7*. Panel B: mRNA expression of genes related to de novo synthesis (*ACC* and *FASN*) and transport (*FABP3*). Panel C: mRNA expression of genes related to triacylglycerol synthesis (*DGAT1*, *DGAT2*, and *PLIN2*). Panel D: mRNA expression of genes related to desaturation (*SCD1*, *FADS1*, and *FADS2*). Values are means ± SEM from 3 individual cultures. * *p* < 0.05 compared with control (Ad-GFP). The data were determined via Student’s t-test. Significance was declared at *p* < 0.05.

Compared with controls (Ad-GFP), the expression of *ELOVL7* after 48 h increased markedly in cells infected with Ad-ELOVL7 (*p* < 0.01, Figure 2A). Overexpression of *ELOVL7* had no significant effect on *ACC*, *FASN,* and *FABP3* (related to FA trafficking) mRNA levels (Figure 2B).

Overexpression of *ELOVL7* significantly decreased the expression of *DGAT2* (*p* < 0.05) and had no effect on *DGAT1* (*p* > 0.05) and *PLIN2* (Figure 2C). Among the detected genes related to FA desaturation, *ELOVL7* overexpression resulted in a lower level of *SCD1* mRNA (*p* < 0.05) and had weak effect on the *FADS1* and *FADS2* (*p* > 0.05) (Figure 2D).

### 3.3. Overexpression of ELOVL7 Altered the Concentration of Long-Chain Unsaturated FA

**Figure 3:**
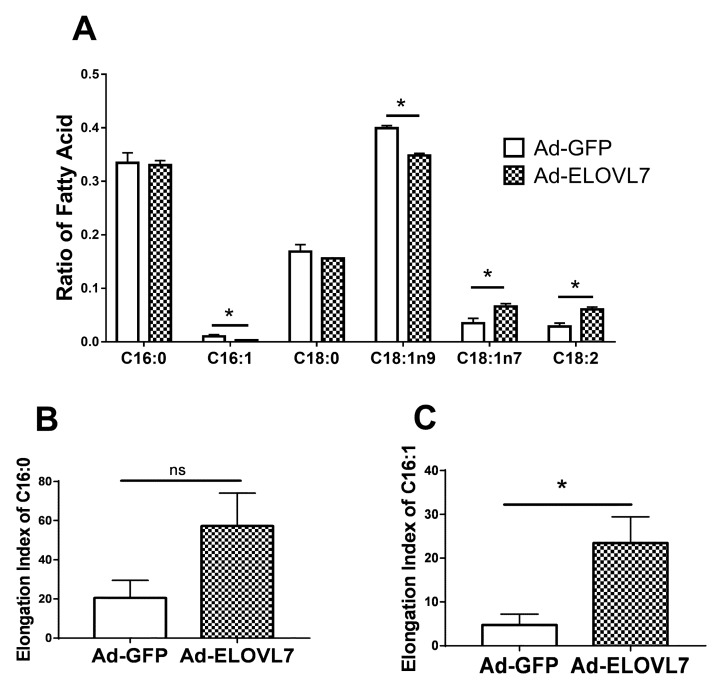
Overexpression of ELOVL7 altered the concentration of C16–C18 carbon fatty acids in goat mammary epithelial cells (GMEC). The GMEC were transfected with Ad-ELOVL7 or Ad-GFP for 48 h and then collected for fatty analysis. Data are reported as ratio of total fatty acids. Values are means ± SEM from 3 individual cultures. The data were determined via Student’s t-test. * *p* < 0.05 compared with control (Ad-GFP). ns represents no significant change compared with control (Ad-GFP).

As shown in Figure 3A, overexpression of *ELOVL7* lowered the concentration of C16:1n7 and C18:1n9 (*p* < 0.05). A significant increase for the concentration of C18:1n7 and C18:2 was observed in the cells overexpressing *ELOVL7*. However, there was no change for the concentration of C16:0 and C18:0. Compared with the control, *ELOVL7* overexpression significantly upregulated the elongation index of C16:1 in GMEC (*p* < 0.05), but had a minor effect on elongation index of C16:0 (Figure 3B,C).

### 3.4. Knockdown of ELOVL7 Altered Genes Related to FA Transport and Desaturation

**Figure 4:**
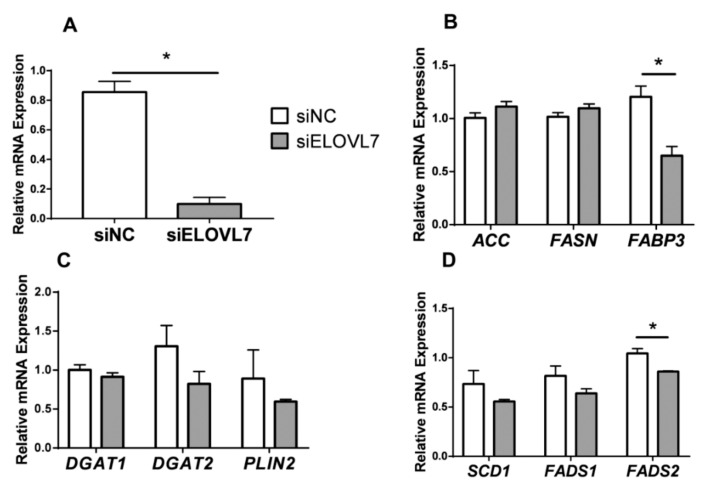
Knockdown of elongation of very-long-chain fatty acid-like fatty acid elongase 7 (*ELOVL7*) altered gene expression. Goat mammary epithelial cells (GMEC) were incubated with siRNA target ELOVL7 (siELOVL7) or negative control (siNC) for 48 h, and then collected for mRNA extraction. Panel A: mRNA expression of ELOVL7. Panel B: mRNA expression of genes related to de novo synthesis (*ACC* and *FASN*) and transport (*FABP3*). Panel C: mRNA expression of genes related to triacylglycerol synthesis (*DGAT1*, *DGAT2*, and *PLIN2*). Panel D: mRNA expression of genes related to desaturation (*SCD1*, *FADS1*, and *FADS2*). Values are means ± SEM from 3 individual cultures. The data were determined via Student’s t-test. * *p* < 0.05 compared with control (siNC).

As shown in Figure 4A, treatment of GMEC with siELOVL7 significantly reduced *ELOVL7* mRNA abundance by 90% (*p* < 0.01). Compared with control (siNC), knockdown of *ELOVL7* downregulated the mRNA level of *FABP3* and *FASD2* (*p* < 0.05) (Figure 4B,D). However, there was no significant changes for *DGAT1*, *DGAT2*, *PLIN2*, *ACC*, *FASN*, *SCD1,* and *FADS1* mRNA in the cells incubated with siELOVL7 (Figure 4B–D).

### 3.5. Knockdown of ELOVL7 Altered the Concentration of Long-Chain Unsaturated Fatty Acids but Not Cellular TAG

**Figure 5:**
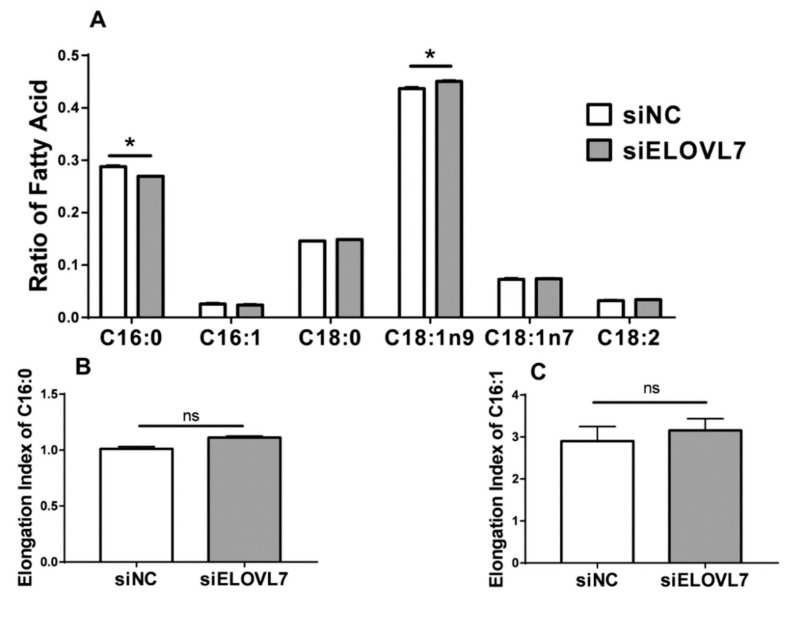
Knockdown of *ELOVL7* altered the concentration of C16–C18 carbon fatty acids in GMEC. Goat mammary epithelial cells (GMEC) were incubated with siRNA target ELOVL7 (siELOVL7) or negative control (siNC) for 48 h and then collected for fatty analysis. Data are reported as ratio of total fatty acids. Values are means ± SEM from 3 individual cultures. The data were determined via Student’s t-test. * *p* < 0.05 compared with control (siNC). ns represents no significant change compared with control (siNC).

Compared with the cells incubated with siNC, the relative concentration of C16:0 in GMEC incubated with siELOVL7 decreased significantly (*p* < 0.05). However, an increase of C18:1n9 concentration was observed in the cells incubated with siELOVL7. Weak changes were observed in the concentration of C16:0, C18:0, vaccenic (C18:1n7), C18:1n9, and linoleic (C18:2) acid after knockdown of *ELOVL7* (Figure 5A). In agreement, compared with the control group (siNC), knockdown of *ELOVL7* did not affect elongation indices of 16-carbon FA (Figure 5B,C). As shown in Figure S1, the concentration of cellular TAG did not change significantly (*p* > 0.05) after overexpression or knockdown of *ELOVL7* in GMEC.

## 4. Discussion

The enzyme ELOVL is rate-limiting for LCFA elongation. It was reported in 293 cells that ELOVL7 plays a role in elongating saturated 16-carbon to 24-carbon FA [12]. However, whether this enzyme plays a role in ruminant MECs has not previously been addressed. The present study delivers new information since we studied LCFA composition and selective regulation of gene expression by switching *ELOVL7* expression in GMEC culture. The present findings highlight a role for ELOVL7 in altering the elongation of 16-carbon to 18-carbon FA.

Previous in vitro elongase activity assays in 293 cells revealed that ELOVL7 was capable of elongating C16:0 and C18:0-CoA [9]. The present data demonstrated that *ELOVL7* overexpression had no significant effect on concentration of C16:0 and C18:0 and indicated that they are therefore not preferential substrates of ELOVL7 in GMEC. The minor change of these two FAs after overexpression of *ELOVL7* agrees with data from prostate cancer cells [11]. The findings in this study, that *ELOVL7* overexpression increased the elongation index of C16:1, are suggestive of a novel role for ELOVL7 in elongating C16:1 to C18:1 in ruminant MEC. However, it is also noteworthy that knockdown of *ELOVL7* had a weak effect on the concentration of C16:1 and C18:1. This response may be caused by “complementary action” among the ELOVL isotypes. This idea is supported by the fact that C16:1 is also a substrate for ELOVL5, and that *ELOVL5* had a higher expression level than *ELOVL7* in GMEC [7]. 

Oleic acid (C18:1n-9) is the predominant 18-carbon FA produced in ruminant MEC, and is synthesized during the desaturation of C18:0. The enzyme SCD1 controls the synthesis of C18:1n9 [24]. The decrease in C18:1n-9 concentration agrees with the lower *SCD1* mRNA level in the cells overexpressing *ELOVL7.* It is noteworthy that overexpression of *ELOVL7* induced an increase of C18:2, which could be synthesized from C18:1 through FADS. Although no change of *FADS1* and *FADS2* mRNA level was observed, we speculate that the FADS protein might be activated by products of ELOVL7. Despite the inability to directly detect the protein activity of FADS in the present study, our speculation is supported by the findings that incubation with siELOVL7 significant decreased *FADS2* expression. The overexpression of *ELOVL7,* causing a lower C18:1n9 concentration and the increase of C18:1n9 upon knockdown of *ELOVL7,* further underscored the role of ELOVL7 in lipid metabolism via its products [3]. These findings emphasize the importance of ELOVL7 in altering the endogenous LCFA concentration in GMEC.

A number of LCFA serve as signaling molecules controlling serial biological functions [3]. In carcinoma cell lines, the finding that knockdown of *ELOVL7* reduced saturated LCFA content and dramatically attenuated cell growth, provides direct evidence for the role of ELOVL7 in controlling cellular signaling [11]. Among the biologically active LCFAs, palmitoleate (C16:1n7) was implicated as a ‘‘lipokine” communicator regulating liver metabolic homeostasis [25]. The alteration of C16:1 in the present study indicated that ELOVL7 may act to control the synthesis of LCFA in GMEC. 

The expression pattern of *ELOVL7* in mammary tissue was similar to that of *ELOVL5*, which also plays a role in elongating C16:1 to C18:1, indicating that the alterations of FA profiles upon knockdown of *ELOVL7* might have been a negative feedback regulation by the products of ELOVL7 activation [7]. Together with the fact that ELOVL7 also catalyzes the elongation of C20, C22, and C24 FA, which are necessary for the cellular membrane composition [15], the higher expression of *ELOVL7* in non-lactating mammary gland tissue suggests that ELOVL7 may participate in mammary gland development through its endogenous products. 

The decrease in *DGAT2* after overexpression of *ELOVL7* is consistent with previous data demonstrating that ELOVL5 activity resulted in *DGAT2* mRNA level [7]. The lack of change in TAG after overexpression or knockdown of *ELOVL7* might be due to insufficient in vitro substrate for GMEC, which was confirmed by the lower expression of *FABP3* induced by knockdown of *ELOVL7*. It is also worth mentioning that alteration of *ELOVL7* in GMEC did not affect *ACC* and *FASN* expression, suggesting there is no interaction between the ELOVL7 (or its products), and FA de novo synthesis in cultured GMEC. The response is consistent with the low level of de novo synthesis activity in culture GMEC. 

In humans, the expression of *ELOVL7* is controlled by the transcription factors, for example, SREBP1 [11,13]. In bovine, the expression of *ELOVL7* is controlled by the activity of SP1 via direct binding on the promoter [14]. In fact, SREBP1 and Sp1 are sensitive to dietary LCFAs in ruminants, indicating that *ELOVL7* could be regulated by dietary LCFA via transcription factors. Whether and how SREBP1, or other dietary LCFA-sensitive transcription factors (e.g., PPAR family), regulate the activity of ELOVL7 remains unclear and, thus, more experiments appear warranted. Collectively, the data from the present study suggest that ELOVL7 plays a regulatory role in controlling long-chain unsaturated FA (LCUFA) elongation in GMEC.

## 5. Conclusions

This study is the first to directly assess the role of ELOVL7 in ruminant MECs. The upregulation of *ELOVL7* increased the concentration of C18:1n7 at the expense of C16:1, whereas knockdown of *ELOVL7* increased the concentration of C18:1n9. The increase of C18:2 at the expense of C18:1n9 in the cells overexpressing *ELOVL7* might be induced by the potential ELOVL7 endogenous products, which serve as cell-signaling molecules. Activation of ELOVL7 decreased the expression of genes related to FA desaturation. In conclusion, at least in vitro, these data demonstrate a direct role for ELOVL7 in modulating LCUFA synthesis in GMEC.

## Supplementary Materials

The following are available online at https://www.mdpi.com/2076-2615/9/6/389/s1, Table S1: Special oligonucleotide sequence of siRNA for ELOVL7. Table S2: Name, accession number, sequences, amplicon length of primer pairs used in the present experiment, efficiency of amplification of PCR, and references. Figure S1: Cellular TAG assays after altering *ELOVL7* expression.
