# Supplementary material for: Fatty Acid Elongase 7 (ELOVL7) Plays a Role in the Synthesis of Long-Chain Unsaturated Fatty Acids in Goat Mammary Epithelial Cells
Source: Animals (Basel). 2019 Jun 25;9(6):389. doi: 10.3390/ani9060389 (PMC6616409; doi:10.3390/ani9060389)
Supplement: Supplementary file 1 [file animals-09-00389-s001.pdf]

animals MDPI

Table 1. Special oligonucleotide sequence of siRNA for *ELOVL7*.

Name	Sequence
siELOVL7-sense	5'- GCACCUGCUGGCUUUAUUATT -3'
siELOVL7-antisense	5'- UAAUAAAGCCAGCAGGUGCTT -3'

Table 2. Name, accession number, sequences, amplicon length of primer pairs used in the present experiment, efficiency of amplification of PCR, and references.

Gene/Acc. #	Primers[1]	Sequence (5' to 3')	bp[2]	Efficiency[3]	Reference.
ACACA XM_005693156.1	F. 3609<br>R.3779	CTCCAACCTCAACCACTACGG<br>GGGGAATCACAGAAGCAGCC	171	2.03	Shi et al., 2013
DGAT1 *XM_005688895.1*	F. 657<br>R.757	CCACTGGGACCTGAGGTGTC<br>GCATCACCACACACCAATTCA	101	1.85	Bionaz and Loor., 2008
DGAT2 *HM566448.1*	F. 192<br>R. 291	CATGTACACATTCTGCACCGATT<br>TGACCTCCTGCCACCTTTCT	100	2.10	Bionaz and Loor., 2008
ELOVL7 *XM_005694673.2*	F. 308<br>R.473	ACTATTCACAGTCGCCTACGG<br>CAGGTCCATGGCATGATGGT	166	2.07	This manuscript
FABP3 *NM_001285701.1*	F. 214<br>R.333	GATGAGACCACGGCAGATG<br>GTCAACTATTTCCCGCACAAG	120	1.92	Shi et al., 2013
FADS1 *EE347846*	F. 552<br>R. 652	GGTGGACTTGGCCTGGATG<br>TGACCATGAAGACAAGCCCC	101	2.18	Bionaz and Loor., 2008
FADS2 *DV895683*	F. 192<br>R. 291	AAAGGGTGCCTCTGCCAACT<br>ACACGTGCAGCATGTTCACA	101	2.06	Bionaz and Loor., 2008
FASN DQ915966.3	F. 6762<br>R.6987	GGGCTCCACCACCGTGTTCCA<br>GCTCTGCTGGGCCTGCAGCTG	226	1.93	Shi et al., 2013
MRPL39 *XM_005674737.1*	F. 370<br>R.470	AGGTTCTCTTTTGTTGGCATCC<br>TTGGTCAGAGCCCCAGAAGT	101	1.94	Kadegowda et al., 2009
PLIN2 *NM_173980*	F. 83<br>R.350	TGGTCTCCTCGGCTTACATC<br>TCTTTTGCCCCAGTCATAGC	268	2.07	Shi et al., 2013
RPS9 *XM_005709411.1*	F.72<br>R.135	CCTCGACCAAGAGCTGAAG<br>CCTCCAGACCTCACGTTTGTTC	64	2.10	Bionaz and Loor., 2007
SCD1 *GU947654*	F. 357<br>R.612	CCATCGCCTGTGGAGTCAC<br>GTCGGATAAATCTAGCGTAGCA	256	1.92	Shi et al., 2013
UXT *XM_005700842.1*	F. 270<br>R.370	TGTGGCCCTTGGATATGGTT<br>GGTTGTCGCTGAGCTCTGTG	101	2.06	Bionaz and Loor., 2007

[1]Primer direction (F-forward, R-reverse) and hybridization position on the sequence.
[2]Amplicon size in base pair (bp)
[3]Efficiency of amplification

Figure 1. Cellular TAG Assays after altering ELOVL7 expression.The protocol for cellular TAG assay was described previously (Kang et al., 2015, Shi et al., 2015b). Total cellular TAG was extracted using the GPO-Trinder triglyceride assay kit (http://www.applygen.com/a/meixueyushenghuaceding/276.html, Applygen Technologies, Beijing, China). The quantification of total cellular TAG was normalized to the cellular protein concentration. Protein concentration of each well was determined using a BCA protein assay kit (Pierce, Thermo fisher Scientific, USA) according to the manufacturer's instructions (https://www.thermofisher.com/order/catalog/product /23225).

A

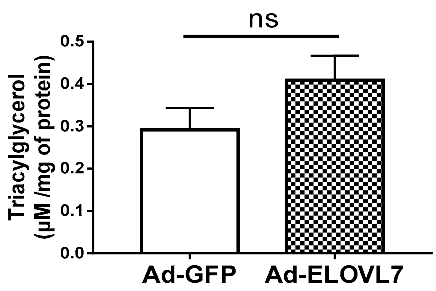

B

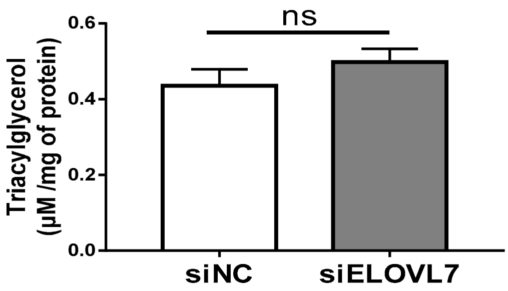

Figure S1. Elongation of very long chain fatty acid-like fatty acid elongase 7 (*ELOVL7*) did not significantly alter the accumulation of cellular triacylglycerol (TAG). The goat mammary epithelial cells (GMEC) were transfected with Ad-ELOVL7 or Ad-GFP or incubated with siRNA target *ELOVL7* (siELOVL7) or negative control (siNC), and collected at 48 h for cellular TAG analysis. Values are means ± SEM from 3 individual cultures. The data were determined via Student's t-test (Ad-ELOVL7 *vs.* Ad-GFP or siELOVL7 *vs.* siNC). ns represents no significant change compared with control.
